# Retrospective View of Four Cases of Cutaneous Manifestations in COVID-19 Patients in an Acute Care Setting

**DOI:** 10.7759/cureus.23003

**Published:** 2022-03-09

**Authors:** Magali Rezende De Carvalho, Elmira Thomas, Barbara Shields-Johnson, Ivan Da Silva

**Affiliations:** 1 Wound and Ostomy Department, Loyola University Medical Center, Maywood, USA; 2 Wound Care Department, The Loretto Hospital, Chicago, IL, USA; 3 Wound Care Department, Loretto Hospital, Chicago, USA; 4 Neurology Department, Rush University Medical Center, Chicago, USA

**Keywords:** pressure injury, pressure ulcer, skin manifestations, wounds and injuries, severe acute respiratory syndrome coronavirus 2, covid-19

## Abstract

Severe acute respiratory syndrome coronavirus 2 (SARS-CoV-2), which is the etiologic agent of coronavirus disease 2019 (COVID-19), causes an excessive inflammatory response and hemostatic abnormalities in the lungs, kidney, and skin. Four patients with COVID-19 admitted to an acute care community hospital developed nonblanchable purpuric macules, patches, and retiform purpura-like lesions at the sacrum, buttocks, lower extremities, and upper back. These lesions can be misdiagnosed as deep tissue pressure injuries. One patient also developed a vesicular-like rash at the upper back and another one developed pernio (chilblains)-like lesions to the third toe of the left foot. Previous studies suggest that the vascular hyperinflammation status and microthrombosis may be responsible for the cutaneous manifestations in patients with SARS-CoV-2. These cutaneous manifestations observed in patients with SARS-CoV-2 may be related to progression of the disease.

## Introduction

Severe acute respiratory syndrome coronavirus 2 (SARS-CoV-2), which is the etiologic agent of coronavirus disease 2019 (COVID-19), causes an excessive inflammatory response and subsequent uncontrolled pulmonary inflammation. Rapid viral replication may be responsible for the massive death of epithelial and endothelial cells that causes vascular leakage [1]. Preliminary reports suggest that in patients severely affected by SARS-CoV-2, hemostatic abnormalities such as thrombocytopenia, increased D-dimer, and fibrinogen serum levels are prevalent along with disseminated intravascular coagulation (DIC) [2-5]. The hypercoagulation state in COVID-19 patients may be involved in microvascular occlusion, which results in stroke and venous thromboembolism [6,7]. Previous studies have reported cutaneous manifestations in COVID-19 patients such as discolorations similar to chilblains on toes, soles, fingers, extremities, and heels, purpuric reticulated eruptions on the lower extremities, and retiform purpura on the buttocks [8,9].

Some of these purpuric lesions may be misdiagnosed as deep tissue pressure injury (DTPI). The National Pressure Injury Advisory Panel (NPIAP) has released a white paper to help wound care clinicians to determine whether the purpuric lesions seen in COVID-19 patients are a DTPI or a skin manifestation of the disease [10].

We present four cases with skin lesions admitted to the COVID unit in a local community hospital between February and May of 2020.

A retrospective review of all patients with diagnosis of COVID-19 who developed any cutaneous manifestation was conducted by the wound care team. Wound care images, notes, and pertinent laboratory results were retrieved from patients’ medical records. Wound care nurses took weekly pictures of all wounds as a standard of care, and verbal consent was obtained prior to taking pictures.

The retrospective case series was reviewed by the Institutional Review Board (IRB) and was considered a quality initiative involving standard of care, and written informed consent was exempted as long as all patient’s data were de-identified.

## Case presentation

Case 1

**Figure 1:**
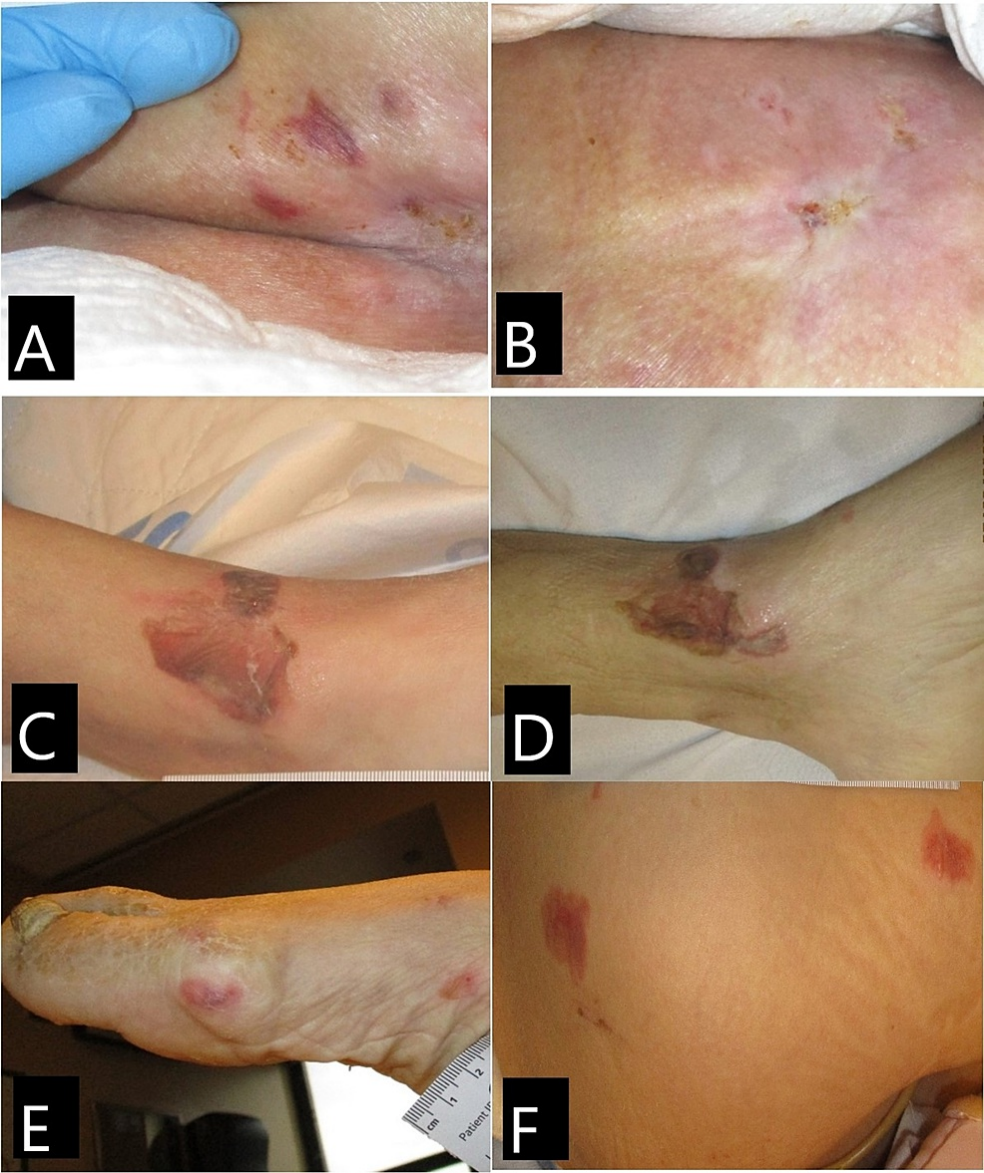
Case 1. Skin lesions. (A) Buttocks on day 1: purpuric patch, nonblanchable. (B) Buttocks on day 10: lesions resolved; note whitish atrophic appearance of the recent healed skin. (C) Left ankle of day 1: serosanguineous-filled bullae. (D) Left ankle on day 10: bullae with no drainage. (E) Right foot on day 1: purpuric nonblanchable patch. (F) Right lateral thigh: nonblanchable purpuric patches and macules.

A female Caucasian patient in her mid 60s was admitted to the hospital due to shortness of breath, persistent fever, dehydration, and hypotension. Past medical history included dementia, schizophrenia, and bipolar disease. She is a nursing home resident and had tested positive for SARS-CoV-2 12 days prior to and again at the time of admission to the hospital. The patient had limited mobility with contracted legs, was bedbound, and had incontinence of the bladder and bowel. She presented to the hospital with multiple lesions at the bilateral lower extremities and sacrum. The sacrum lesion measured (Figure 1B). Left ankle lesion presented as serosanguineous-filled bullae, measuring 4 x 4 cm (Figure 1C). On day 10, the bullae lesion did not presented any drainage, and no tenderness was noted (Figure 1D). Right foot showed multiple nonblanchable macules and patches (Figure 1E). Right lateral thigh presented with two non-blanchable purpuric patches, measuring 3 x 3 cm and 2.5 x 2 cm (Figure 1F). On day 10, thigh lesions have reduced in size and foot lesions had resolved. The patient was placed on a non-powered reactive air support surface, with foam heel protectors, and was turned and repositioned every two hours to offload pressure points, prevent worsening of the wounds, and avoid pressure injuries. She was safely discharged back to her nursing home on day 10.

During hospital stay, the patient’s renal function remained stable, but signs of systemic inflammation were present with a significantly high serum level of C-reactive protein.

During hospitalization, medications included azithromycin, enoxaparin sodium, mirtazapine, atorvastatin, valproic acid, piperacillin, and midodrine.

Case 2

**Figure 2:**
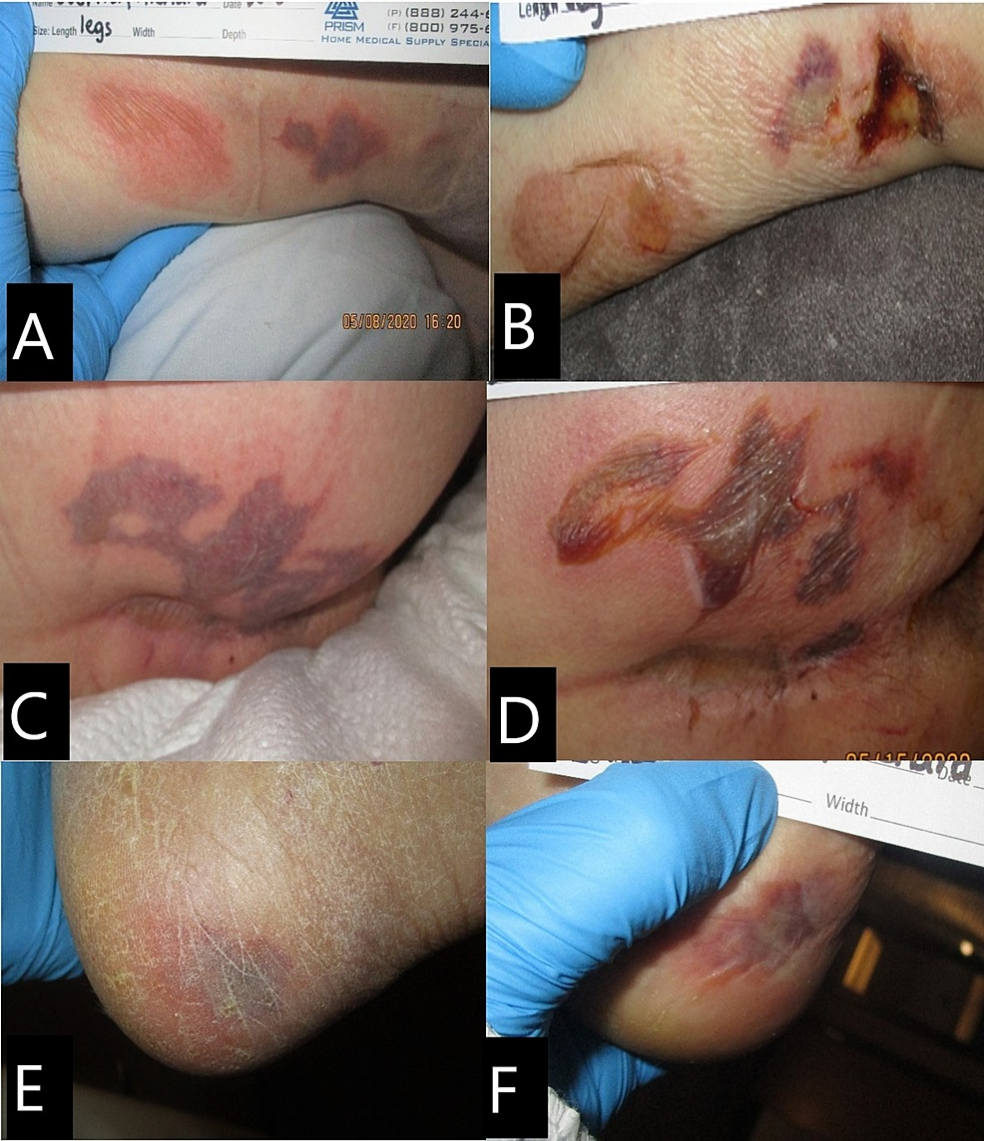
Case 2. Skin lesions. (A) Right lower leg on day 10: purpuric nonblanchable patch. (B) Right lower leg on day 14: serous-filled bullae with some necrotic tissue. (C) Right buttock on day 10: purpuric nonblanchable patch resembling retiform purpura. (D) Right buttock on day 14: lesion evolved to a serosanguineous-filled bullae (E) Left lateral heel on day 10: purpuric nonblanchable patch. (F) Left lateral heel on day 14: purpuric nonblanchable patch.

A male Caucasian patient in the late 60s admitted to the hospital due to cough and fever and tested positive for SARS-CoV-2 at admission. Past medical history indicated chronic obstructive pulmonary disease (COPD), lung cancer, hypertension, and schizophrenia. He was intubated on day 2 due to acute respiratory distress. No wounds were noted at admission. A non-powered reactive air support surface as well as heel protectors were applied, and the patient was turned and repositioned every two hours. He soon became febrile, and developed generalized edema, hypoxemia, and hypotension requiring use of vasopressors. On day 10, multiple skin lesions were observed on the buttocks, legs, and heels. The right and left legs developed purpuric nonblanchable patches, and the right leg lesions measured 5 x 4 cm and 5 x 3 cm (Figure 2A); on day 14, lesions evolved to serous-filled bullae with some necrotic tissue (Figure 2B). Right buttock and gluteal cleft developed a purpuric nonblanchable patch measuring 9 x 3 cm, resembling retiform purpura that evolved to a bullae on day 14 (Figures 2C, 2D). Right lateral heel developed another purpuric patch, nonblanchable, no tenderness or induration was noted, and lesion measured 2 x 2 cm (Figure 2E); on day 14, lesion appeared darker, but there are no changes to palpation (Figure 2F). All skin lesions were cleaned and covered with silicone foam dressing. The patient passed away on day 20.

Of note, this patient developed a marked systemic inflammatory response, evidenced by large elevations of serum C-reactive protein levels and increase in pro-coagulation markers, including high serum fibrinogen and D-dimer concentrations. The patient’s renal function remained grossly stable.

The patient's medications in use during admission were morphine, propofol, ceftriaxone, meropenem, vancomycin, norepinephrine, enoxaparin, and fentanyl during hospitalization.

Case 3

**Figure 3:**
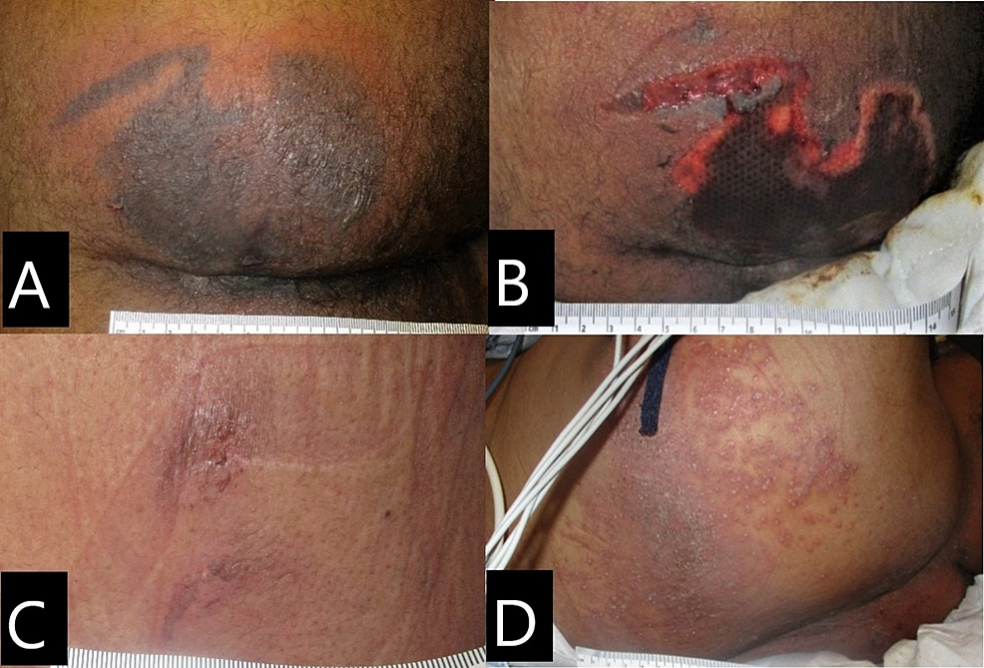
Case 3. Skin lesions. (A) Right buttock on day 6: purpuric patch lesion with periwound blanchable erythema. (B) Right buttock on day 14: necrotic wound base. (C) Upper back on day 6: purpuric patch with ruptured blisters. (D) Upper back on day 14: vesicular-like rash.

A male African American patient in early 50s, resident of a nursing home, was admitted to the hospital due to persistent fever and urinary tract infection. He tested positive for SARS-CoV-2 upon admission. Past medical history indicated cerebrovascular accident (CVA), human immunodeficiency virus (HIV), COPD, diabetes mellitus (DM), morbid obesity, and schizoaffective disorder. The patient had no wounds at admission and was febrile and confused but able to interact with the staff, feed himself, and turn and reposition in bed. He remained febrile for the next few days despite medication and non-pharmacologic measures for fever control. On day 5, his fever was still uncontrolled, he became hypotensive, and oxygen levels dropped requiring a non-rebreather mask at 5 L/min of O2. He was responsive to painful stimuli only. He was then placed on a non-powered reactive air support surface with foam heel protectors and was turned and repositioned every two hours. On day 6, the patient developed a purpuric nonblanchable patch on the right buttock (Figure 3A). Right buttock had a purpuric patch that was resembling retiform purpura, the periwound skin presented with blanchable erythema, no tenderness or induration was noted, and the lesion measured 13 x 11 cm. On day 14, buttock lesion had evolved with necrotic tissue, with stable eschar at wound base, warm and tender to touch, with heavy serosanguineous exudate, and no foul odor (Figure 3B). The patient also developed two purpuric patches with partially ruptured blisters on his upper back on day 10; lesions were measuring 2.5 x 1 cm and 1.5 x 0.8 cm (Figure 3C). On day 14, a vesicular-like rash on the upper back was noted (Figure 3D). The patient was able to transition to a high-flow mask at 10 L/min; although awake, he had low-grade fever and was administered vasopressor when his blood pressure dropped. On day 21, the upper back rash had improved, and the buttock wound showed no changes. The patient was safely discharged on day 24 back to his nursing home.

Regarding laboratory serum monitoring, this patient developed a significant systemic inflammatory response, with substantial elevations of serum C-reactive protein levels, as well as a possible increase in pro-coagulation, including elevations of serum fibrinogen and D-dimer concentrations. The patient’s renal function remained stable throughout hospital stay.

The patient's medication list during hospital admission included vancomycin, ritonavir, cefepime, norepinephrine, hydroxychloroquine, doxycycline, enoxaparin, ibuprofen, piperacillin sodium, acetaminophen, ondansetron, zinc sulfate, emtricitabine, and ceftriaxone during hospitalization.

Case 4

**Figure 4:**
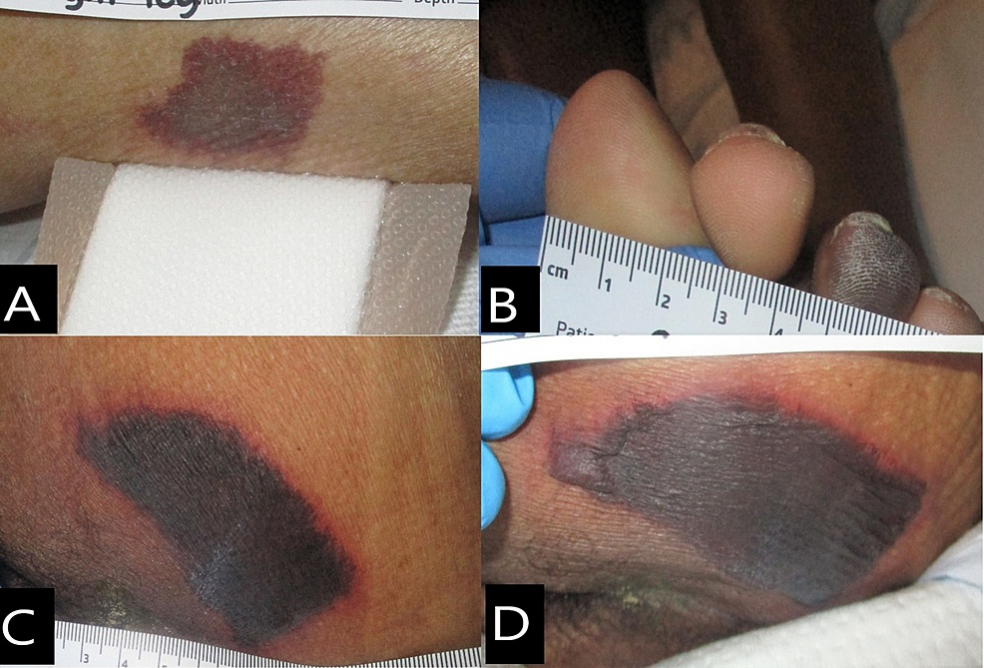
Case 4. Skin lesions. (A) Right lower leg on day 10: purpuric nonblanchable patch. (B) Third digit of left foot on day 10: pernio-like lesion. (C) Left buttock on day 14: necrotic patch with nonblanchable erythema at periwound skin. (D) Left buttock on day 19: necrotic patch with bullae at edges.

A male African American patient in his late 50s was admitted to the hospital due to fever and cough. His past medical history indicated hypertension. The COVID-19 test done at admission was negative. The test was repeated on day 4 and came back positive. The patient had fever, watery diarrhea, dry cough, and labored breathing on 4L/min of O_2_. On day 2, the patient developed acute kidney failure and acute hypoxic respiratory failure. X-ray showed atypical pneumonia. On day 5, he remained febrile and severely lethargic with 80% O_2_ saturation. He was placed on a 100% non-rebreather oxygen mask. On day 7, he was still febrile, with tachycardia, tachypnea, and hypotension; he was in acute respiratory distress requiring intubation and vasopressors. A non-powered reactive air support surface and foam heel protectors were applied, and he was turned and repositioned every two hours. The patient developed septic shock and cytokine storm. He was started hemodialysis on day 10 and was still febrile, and two skin lesions were noted. The right lateral lower leg had a purpuric nonblanchable patch that measured 4 x 2 cm (Figure 4A); the purpuric patch had no induration and Was not warm to touch. The third digit on the left foot developed a pernio (chilblain)-like lesion (Figure 4B). On day 11, he was afebrile and off the vasopressors. His buttock was assessed, which showed a necrotic lesion, with purpuric patch and blanchable erythema at periwound skin, and lesion measured 8 x 6 cm on day 14 (Figure 4C). The patient started improving and was extubated on day 17. He was still tachycardic on metoprolol and hemodialysis. On day 19, right leg lesion and third digit lesion resolved. The left buttock remained the same size, but was evolving into a serous-filled bullae (Figure 4D). He was discharged to a long-term rehabilitation facility on day 20.

Finally, the patient showed signs of marked systemic inflammation, with severe elevation of serum levels of C-reactive protein. His blood levels of fibrinogen suggested a possible shift to a pro-coagulant state, and his renal function deteriorated to the point of requiring renal replacement therapy.

Metoprolol, methylprednisolone, minocycline, cefepime, labetalol, heparin, propofol, fentanyl, diltiazem, norepinephrine, meropenem, vasopressin, piperacillin, ceftriaxone, and azithromycin were given to the patient during hospitalization.

## Discussion

The cutaneous manifestations seen in acute care and outpatient COVID-19 patients are described herein. The most common cutaneous lesions described in patients with COVID-19 are acral, chilblain-like, urticaria-like, vesicular chickenpox-like, and maculopapular lesions [11].

Although some theories have been presented to explain the course of the disease, the mechanisms of SARS-CoV-2 cutaneous manifestations are not well defined. One theory involves the participation of the angiotensin-converting enzyme 2 (ACE2) as a possible functional receptor for SARS-CoV-1 (2003-2004 outbreak) as well as for SARS-CoV-2 (2019-2020 outbreak) [12,13]. ACE2 can be found in vascular endothelial cells, smooth muscle cells, and basal epidermal layer of the skin [13]. SARS-CoV-2 binds to ACE2 as a cellular receptor, becomes internalized into the cytoplasm, and forms a phagosome. These events could contribute to the amplification of the inflammatory response and lymphocytic vasculitis [13,14]. The activation of ACE2 engenders antithrombogenic effects; therefore, when infected by SARS-CoV-2, the regular functions of ACE2 become impaired and it loses the ability to prevent thrombosis [15].

This case series reports different skin manifestations in patients diagnosed with COVID-19 disease. We observed purpuric nonblanchable macules and patches, pernio-like lesions, retiform purpura-like lesions, and a vesicular-like rash.

At first assessment, the wound care team described the purpuric macule, patches, and bullae lesions as DTPI, which is defined by NPIAP as an “intact or non-intact skin with localized area of persistent nonblanchable deep red, maroon, purple discoloration or epidermal separation revealing a dark wound bed or blood filled blister” [16]. However, with the rapid evolution of these purpuric discolorations and an odd increase in the incidence of DTPIs among COVID-19 patients, the team decided to take a closer look at the lesions and search for some answers in the literature.

All pressure injuries acquired after a patient is admitted to the hospital are considered hospital-acquired pressure injury (HAPI). HAPI is recognized as a financial burden to the hospital and is a quality indicator for Magnet recognition. Therefore, it is important to differentiate between COVID-19-related cutaneous lesions and pressure injuries.

The patient cited in case 3 (Figure 3) was obese and had a history of psychiatric disorder; when his alertness increased, the patient became more agitated and non-compliant with the repositioning schedule. Therefore, pressure and shear probably played an important role in the outcome of the buttocks wound. We believe that the cause of this wound was microthrombosis related to COVID-19 disease, but the worsening of the wound was related to pressure.

The vascular hyperinflammation status and microthrombosis could be responsible for some of the cutaneous manifestations observed in patients with COVID-19 [10].

One study had performed a biopsy of the skin lesion and found an extensive pattern of pauci-inflammatory vascular thrombosis with endothelial cell injury and microvascular deposits of the complements C5b-9 and C4d [9]. These findings suggest that the extensive deposition of C5b-9 and C4d could be responsible for the microvascular endothelial cell injury leading to microthrombosis observed at the skin lesions as well as in the lungs of patients with SARS-CoV-2 [9]. Another report from Cleveland Clinic described two cases presenting a morbilliform rash, acral purpura reminiscent of perniosis, and a purpuric plaque with retiform/livedoid borders on buttocks with biopsy showing features consistent with a thrombotic vasculopathy and probable related to COVID-19 [17]. A similar retiform/livedoid border was noted around the lesions on buttocks in the patients described on cases 3 and 4.

Unfortunately, biopsy of the lesions, and D-dimer and fibrinogen serum tests were not performed consistently due to the limited resources available at the local community hospital. Considering the beginning of the pandemic, lack of knowledge of the disease at that time, the high volume of critically ill patients, and limited resources, dermatological consults were not available at the hospital at that moment; therefore, no other differential diagnosis were explored.

These cutaneous manifestations observed in patients with COVID-19 may be related to the progression of disease. Further investigation is needed to fully elucidate the pathophysiology of these cutaneous manifestations observed in these patients.

## Conclusions

COVID-19 disease appears to affect the skin and may be responsible for the microvascular thrombosis that results in lesions with purpuric patch/macules or retiform purpura-like lesions in acute care patients. The lesions may mimic the appearance of DTPI. These lesions should be carefully evaluated by the healthcare providers to safely rule out pressure injury and other disorders with similar presentation. The hyperinflammatory state, which is thought to be responsible for the rash observed in one of the patients, may contribute to the microthrombosis observed in SARS-CoV-2 positive patients. Further investigation is needed to fully elucidate the pathophysiology of these cutaneous manifestations observed in patients diagnosed with COVID-19.
